# CRISPR/Cas9-induced DNA breaks trigger crossover, chromosomal loss, and chromothripsis-like rearrangements

**DOI:** 10.1093/plcell/koad209

**Published:** 2023-07-27

**Authors:** Aviva Samach, Fabrizio Mafessoni, Or Gross, Cathy Melamed-Bessudo, Shdema Filler-Hayut, Tal Dahan-Meir, Ziva Amsellem, Wojciech P Pawlowski, Avraham A Levy

**Affiliations:** Department of Plant and Environmental Sciences, The Weizmann Institute of Science, Rehovot 7610001,Israel; Department of Plant and Environmental Sciences, The Weizmann Institute of Science, Rehovot 7610001,Israel; Department of Plant and Environmental Sciences, The Weizmann Institute of Science, Rehovot 7610001,Israel; Department of Plant and Environmental Sciences, The Weizmann Institute of Science, Rehovot 7610001,Israel; Department of Plant and Environmental Sciences, The Weizmann Institute of Science, Rehovot 7610001,Israel; Department of Plant and Environmental Sciences, The Weizmann Institute of Science, Rehovot 7610001,Israel; Department of Plant and Environmental Sciences, The Weizmann Institute of Science, Rehovot 7610001,Israel; School of Integrative Plant Science, Cornell University, Ithaca, NY 14853,USA; Department of Plant and Environmental Sciences, The Weizmann Institute of Science, Rehovot 7610001,Israel

## Abstract

DNA double-stranded breaks (DSBs) generated by the Cas9 nuclease are commonly repaired via nonhomologous end-joining (NHEJ) or homologous recombination (HR). However, little is known about unrepaired DSBs and the type of damage they trigger in plants. We designed an assay that detects loss of heterozygosity (LOH) in somatic cells, enabling the study of a broad range of DSB-induced genomic events. The system relies on a mapped phenotypic marker which produces a light purple color (betalain pigment) in all plant tissues. Plants with sectors lacking the *Betalain* marker upon DSB induction between the marker and the centromere were tested for LOH events. Using this assay, we detected a tomato (*Solanum lycopersicum*) flower with a twin yellow and dark purple sector, corresponding to a germinally transmitted somatic crossover event. We also identified instances of small deletions of genomic regions spanning the T-DNA and whole chromosome loss. In addition, we show that major chromosomal rearrangements including loss of large fragments, inversions, and translocations were clearly associated with the CRISPR-induced DSB. Detailed characterization of complex rearrangements by whole-genome sequencing and molecular and cytological analyses supports a model in which a breakage–fusion–bridge cycle followed by chromothripsis-like rearrangements had been induced. Our LOH assay provides a tool for precise breeding via targeted crossover detection. It also uncovers CRISPR-mediated chromothripsis-like events in plants.

## Teaser

An assay to detect loss of heterozygosity shows various outcomes for DNA double-stranded break repair, such as crossing over, and aberrant repair leading to multiple chromosomal rearrangements.

**IN A NUTSHELL**

**Background:** Plant breeding is the art of combining desirable traits from parental genomes. This normally occurs during meiosis through crossover, the exchange of chromosomal segments between homologous chromosomes. So far it has not been possible to target the precise breakpoints of crossovers at meiosis. Somatic crossover is rare, but we previously showed that it can be stimulated by a DNA double-strand break (DSB) and can be transmitted to the next generation. This was shown in tomatoes (*Solanum lycopersicum*) at specific fruit color loci, but a more general system is needed to become a useful tool for precise breeding.

**Question:** To better understand somatic DSB repair via homologous recombination, we developed an assay enabling visualization of somatic crossover via segregation of a transgenic purple color marker (the betalain pigment), at a broad range of loci. Through this process, heterozygous tissues become homozygous, forming wild-type or dark purple sectors in tomato leaves, flowers, or fruits.

**Findings:** We confirmed that somatic crossover can be detected visually at a CRISPR-mediated DSB site, opening the prospect for precise breeding in crops. Moreover, we showed that loss of heterozygosity could be due to major chromosomal rearrangements triggered by defective repair of the DSB. Plants where this occurred contained large deletions and translocations and were sterile, showing micronuclei as well as bridges in dicentric chromosomes, at meiosis, as described in McClintock's breakage–fusion–bridge cycle triggered by transposons. This genomic reshuffling is similar to chromothripsis in mammalian cells. Both crossover and chromothripsis events were rare but could be detected thanks to the visual assay.

**Next steps:** It will be important to better understand what determines the fate of a DSB, when repair leads to crossover or, alternatively, to chromothripsis. The new assay will also enable to study how targeted somatic crossover can become more efficient for breeding applications.

## Introduction

DNA double-stranded breaks (DSBs) can be repaired by nonhomologous end-joining (NHEJ) or by homologous recombination (HR). Error-prone NHEJ can generate small insertions or deletions (Indels) at the DSB site (Gorbunova and Levy 1997). The outcomes of HR vary according to the homologous partners: recombination between repeats in cis can lead to deletions in the case of direct repeats or inversions in the case of inverted repeats (Lupski 1998). HR between ectopic repeats (Shalev and Levy 1997; Puchta 1999) can lead to translocations. DSBs were also shown to induce crossovers (COs) between sister chromatids or homologous chromosomes in somatic cells (Molinier et al. 2004).

The CRISPR-Cas9 system enables analyzing the DSB repair process at endogenous loci and in a targeted manner, becoming an invaluable tool for precise breeding (Barrangou and Doudna 2016). It is now possible to perform targeted mutagenesis, or when multiple breaks are induced, NHEJ enables precise chromosome engineering through deletions, inversions, or translocations of large chromosomal segments (Beying et al. 2020; Schmidt et al. 2020). CRISPR-induced HR-mediated repair enabled enhancing gene replacement frequencies (Baltes et al. 2014; Schiml et al. 2014; Dahan-Meir et al. 2018) or achieving targeted COs or gene conversions (Filler-Hayut et al. 2017, 2021; Ben Shlush et al. 2020). However, in the absence of selection, rates of targeted CO are quite low (Filler-Hayut et al. 2021), and in tomato (*Solanum lycopersicum*), targeted CO events were so far identified only by using visual fruit color markers (Filler-Hayut et al. 2017; Ben Shlush et al. 2020).

While the promises of genome editing for precise plant breeding are immense, there are still many challenges. Repair is not always efficient, and unrepaired DSBs can have deleterious consequences that have not been carefully analyzed in the CRISPR context. Recent studies in mammalian cells have demonstrated that CRISPR-Cas9 can induce loss of heterozygosity (LOH) as a result of loss of segments, arms, or whole chromosomes, as well as a cascade of chromosomal rearrangements following cell divisions (Zuccaro et al. 2020; Alanis-Lobato et al. 2021; Leibowitz et al. 2021). These rearrangements are similar to those found in cancer and can have unintended consequences on the edited genomes and cells. A few percent of the cells that underwent Cas9-induced DSBs exhibit chromosome bridges and, later, micronuclei (Leibowitz et al. 2021).

This is consistent with a model in which chromosomal rearrangements occur through breakage–fusion–bridge cycles (BFBCs). Such cycles were first discovered in the pioneering work of Barbara McClintock, who studied the fate of chromosomes broken through irradiation or transposable element activities in maize (*Zea mays*). She proposed that sister chromatids of chromosomes with unrepaired broken ends become joined via what we call now NHEJ, generating a dicentric chromosome that can be broken at anaphase when the 2 centromeres are pulled to the opposite poles (McClintock 1941). This process can repeat itself, triggering a cycle of random breaks between the centromeres and subsequent repair, leading to a BFBC. The outcomes of this process can be a range of chromosomal rearrangements, including deletions, duplication, and inversion of large chromosomal segments or whole chromosome loss. In cancer cells, the BFBC was shown to further trigger a series of catastrophic events, leading to chromothripsis: for example, an acentric segment can be excluded from the nucleus, forming a micronucleus, then the micronucleus DNA content can undergo fragmentation, and the resulting fragments can reintegrate into the genome, causing additional chromosome rearrangements (Kwon et al. 2020; Ostapińska et al. 2022).

In plants, it is unclear whether CRISPR-Cas9-induced DSBs can induce a BFBC. In addition, chromothripsis has received little attention so far, except for findings in *Arabidopsis* (*Arabidopsis thaliana*), suggesting that genome elimination occurring in hybrids containing an altered centromeric histone CENH3 was reminiscent of chromothripsis (Tan et al. 2015; Henry et al. 2018).

In this work, we developed an assay that enables visual detection of LOH using a hemizygous transgenic *Betalain* (*Bet*) marker. DSB-induced LOH, due to somatic loss or HR of chromosomal segments carrying the *Bet* marker, could be seen as green sectors in a purple background or as twin sectors (green and dark purple) in leaves. Betalains are purple pigments found in flowers, leaves, roots, and fruits of plants of most families of the Caryophyllales (Strack et al. 2003). Three enzymes are essential in the biosynthesis pathway of betalains, and a cassette containing genes encoding them was inserted into several plant species, including tomato (Polturak et al. 2016). The genomic integration site of the T-DNA construct containing the 3 genes of the betalain purple pigment biosynthetic pathway (here termed the *Bet* marker) was identified, and the CRISPR-Cas9 system was used to induce DSBs between the marker and the centromere. Somatic sectors corresponding to putative deletion or HR events were segregated or regenerated into whole plants and analyzed at the molecular level, identifying a flower somatic sector. These observations validate the concept that rare somatic CO events can be visually detected at a desired locus, using a nearby transgenic marker. In addition, the LOH assay enabled us to detect unrepaired DSB events, leading to the loss of the whole chromosome or chromosome segments carrying the T-DNA marker. We show that these events can be explained by the induction of a BFBC leading to chromothripsis.

## Results

### A general system for DSB-induced LOH detection using the *Bet* marker and CRISPR-Cas9

**Figure 1.**
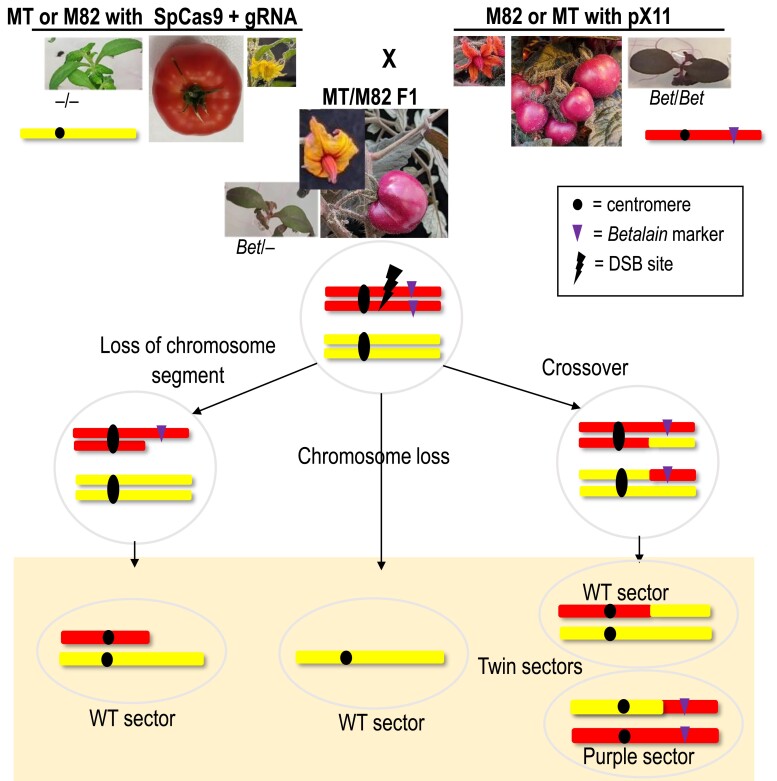
The Betalain visual assay for LOH via deletion or CO. Green MT/M82 SpCas9 + gRNA is crossed with purple M82/MT with *Bet* expression T-DNA (pX11) to generate an F1 hybrid. The light purple F1 is hemizygous for the pX11 T-DNA (*Bet*/−). The chromosome on MT/M82 SpCas9 + gRNA target line has mutations at the gRNA target site and cannot be broken. The target chromosome in M82/MT pX11 plants can undergo a DNA DSB at the target site. The DSB could be perfectly repaired or repaired with small Indels by NHEJ. In these cases, the plants will remain light purple and will not be selected. LOH outcomes that can generate a WT phenotype (green leaf sector, yellow flower sector, or red fruit sector without betalain) are shown in the bottom box. From left to right: loss of DSB distal fragment or part of it that contains the pX11 T-DNA, whole chromosome loss, or CO that can generate a twin sector (WT color adjacent to dark purple). Black circles represent the centromere. The purple triangle represents the T-DNA containing the *Betalain* marker and the black lightning bolt represents the site of CRISPR-Cas9 break.

In order to characterize DSB-induced LOH events and understand their underlying cause, we have developed a system based on a known-location dominant visual genetic marker (termed *Bet* and resulting in production of the pigment betalain) in a heterozygous background (Fig. 1). A DSB induced in somatic cells anywhere between the marker and the centromere can be repaired by NHEJ, with no phenotypic consequences, or by HR, which, in case of a CO, can yield a twin sector, i.e. a wild-type (WT) color transgene-free sector (−/−) and a transgene–homozygous dark purple sector (*Bet*/*Bet*) in the light purple hemizygote (*Bet*/−) background. If the DSB is unrepaired, LOH can also be detected as loss of the phenotypic marker.

**Figure 2.**
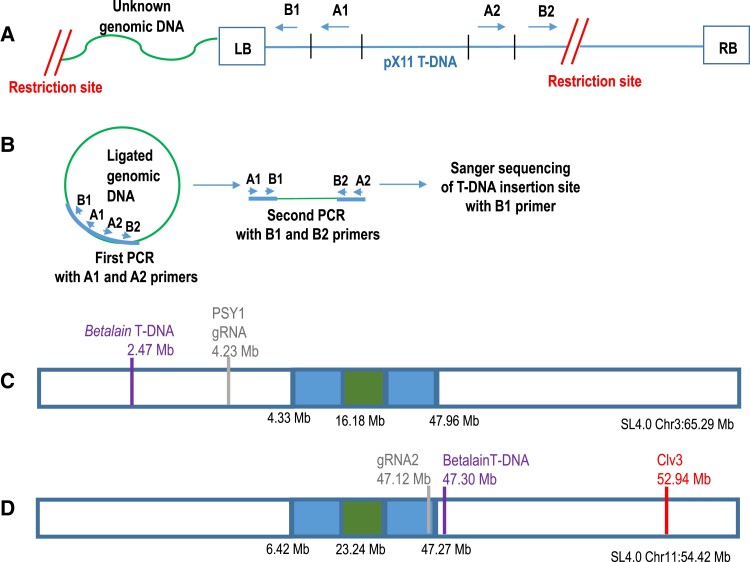
Sequencing pX11 T-DNA insertion sites by inverse PCR. **A)** Plant DNA is restricted with restriction enzyme (PstI or HindIII), followed by self-ligation. **B)** PCR amplification with 2 sets of nested primers, A1 and A2 and then B1 and B2, followed by Sanger sequencing. This enabled the identification of the genomic sequence at the junctions of the LB of the T-DNA. **C)** Illustration of SL4.0 Chromosome 3 with coordinates of the *PSY1* gRNA DSB (light gray line) target and the mapped pX11 T-DNA integration site in M82 (purple line). **D)** Illustration of SL4.0 Chromosome 11 with coordinates of the gRNA2 DSB target (light gray line), the mapped pX11 T-DNA integration site in MT (purple line), and the *CLV3* locus (red line). Centromere, green box; heterochromatic region, blue box; euchromatic region, white box. Estimated SL4.0 coordinates of centromere, heterochromatic, and euchromatic regions are given below in megabases. The complete size in megabases for each chromosome is shown underneath the chromosome illustration.

The T-DNA construct (pX11) containing 3 betalain biosynthesis genes was previously transformed into tomato Micro-Tom (MT) (Polturak et al. 2017). Seeds were kindly provided by the Aharoni Lab. We also generated new lines carrying pX11 in the M82 background. We mapped the T-DNA insertion site using inverse PCR (Fig. 2). Primers were designed for the left border (LB) region of the pX11 cassette (Supplemental Table S1). DNA from pX11 homozygous lines in the M82 and MT backgrounds was extracted and then digested using PstI and HindIII restriction enzymes, respectively (Fig. 2A). The digested DNA was then self-ligated and subjected to 2 rounds of PCR amplification with nested primers. PCR products were Sanger-sequenced using the LB primer (Fig. 2B) (Thomas et al. 1994). Sequence regions that did not align with the pX11 sequence were then compared using BLAST against the tomato genome, which revealed the location of the junctions between the pX11 T-DNA and the M82 or MT genomes. Primers were then designed from both sides of the T-DNA insertion site to verify both the LB and RB junctions. This procedure confirmed the pX11 integration sites. In M82, the pX11 integration site was found on the short arm of Chromosome 3, SL4.0ch3:2,475,240, downstream of Solyc03g007960 (Fig. 2C). In MT, pX11 integration was on the long arm of Chromosome 11, SL4.0ch11:47,305,369, upstream of Solyc11g062370 (Fig. 2D). We named this marker locus *Bet*.

In order to identify LOH events, the system included the mapped *Bet* marker (Fig. 2) in the F1 hybrid (MT × M82-pX11) and (M82 × MT-pX11) backgrounds. LOH could be identified as dark purple *Bet/Bet*, or WT transgene-free (green in leaves, yellow in flowers, or red in fruit) somatic sectors in the hemizygote light purple *Bet/*− background (Fig. 1). Moreover, single-nucleotide polymorphisms (SNPs) differentiating between the parental lines could be used for genotyping at the whole chromosome scale. Targeted DSBs were induced with CRISPR-Cas9 between the centromere and the T-DNA. MT and M82 plants containing the SpCas9 + gRNA, both controlled by constitutive promoters, were crossed to M82 and MT pX11 plants, respectively (Fig. 1).

On Chromosome 3, we designed the gRNA to target a euchromatic region (Demirci et al. 2017) at position SL4.0ch3:4,236,695 in Exon 4 of phytoene synthase (*PSY1*), at the distance of 1,761,455 bp from the M82 pX11 marker proximal to the centromere (Fig. 2C; Supplemental Table S2). A transgenic MT plant carrying SpCas9 and *PSY1* gRNA produced an NHEJ footprint at the DSB target of GCT deletion (−GCT) in 50% of the reads and G deletion (−G) in 50% of the reads (Dahan-Meir et al. 2018). This represents 1 chromosome with (−GCT) and 1 chromosome with (−G) footprints. Progeny of this MT plant carrying SpCas9 and *PSY1* gRNA, produced by selfing, had the same NHEJ footprints at the *PSY1* gRNA DSB target site (100% [−GCT], 100% [−G], or 50% [−GCT]/50% [−G]), indicating no further DSB formation. The transgenic MT plant carrying SpCas9 and *PSY1* gRNA was used for crossing with M82 pX11. Since the *PSY1* gRNA target on the MT Chromosome 3 was mutated, only the M82 pX11 Chromosome 3 could be cleaved by Cas9 in the F1 plants.

On Chromosome 11, we targeted the gRNA to a heterochromatic region (Demirci et al. 2017) at position SL4.0ch11:47,124,456 (gRNA2), between 2 gene promoters at the distance of 180,913 bp from the MT pX11 marker proximal to the centromere (Fig. 2D; Supplemental Table S2). The transgenic M82 plant with SpCas9 and gRNA2 gave an NHEJ footprint at the DSB target of +T insertion (+T) in 100% of the reads. Progeny of this plant produced by selfing had the same NHEJ footprint at the Chromosome 11 gRNA2 target site (100% [+T]), indicating no further DSB formation. The transgenic M82 plant with SpCas9 and gRNA2 was used for crossing with MT pX11. Since the gRNA2 target of the M82 Chromosome 11 was mutated, only the MT pX11 Chromosome 11 could be cleaved by Cas9 in the F1 plants.

### Screening for twin sectors as putative CO events

Twin sectors, consisting of a dark purple sector (*Bet/Bet*) adjacent to a WT sector (*−/−*) in the light purple background (*Bet/−*) can represent putative somatic CO events originating from a reciprocal exchange between chromatids of homologous chromosomes as shown in Fig. 1. To induce and visually identify such somatic events, we screened (*Bet/−*) plants where DSBs were induced between the *Bet* markers mapped on Chromosomes 3 and 11 and centromeres as shown in Fig. 2, C and D, respectively.

**Figure 3.**
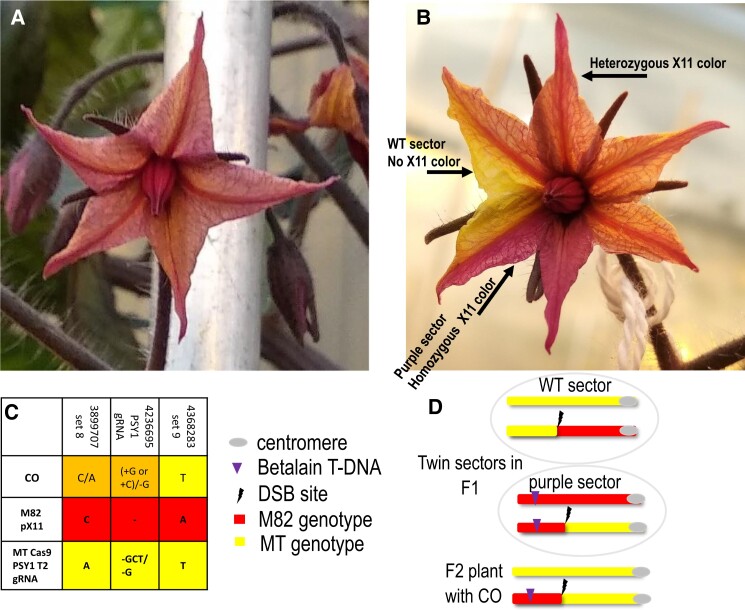
Detection of a targeted somatic CO event on Chromosome 3 seen as a twin sector. A DSB was induced by the *PSY1* gRNA on Chromosome 3, between the centromere and the *Betalain* marker. **A)** Tomato flower of F1 plant ([MT SpCas9 + *PSY1* gRNA]/[M82 pX11 on chromosome 3]) with uniform light purple color phenotype. **B)** Tomato flower of F1 plant ([MT SpCas9 + *PSY1* gRNA]/[M82 pX11 on Chromosome 3]) with chimeric twin sector color phenotypes WT yellow (no transgene) next to a dark purple sector (*Bet*/*Bet*). One F2 progeny out of 6 in the fruit generated from this flower was a CO event. **C)** Sanger sequencing results of 3 SNP sets on Chromosome 3: (i) Set 8 upstream to the DSB site proximal to the telomere side; (ii) SpCas9 + *PSY1* gRNA, generated SNP at the DSB site in the MT SpCas9 + PSY1 gRNA background; (iii) Set 9 downstream to the DSB site proximal to the centromere side. In the CO event F2 generated from the twin sector flower in Panel **B)**, we see a transition from heterozygous MT/M82 SNPs in Set 8 and *PSY1* gRNA to homozygous MT SNPs in Set 9. Orange highlight, heterozygous MT/M82 SNPs; red highlight, homozygous M82 SNPs; yellow highlight, homozygous MT SNPs. **D)** Scheme of the recombinant Chromosome 3 generated by SpCas9-induced somatic CO and detected by following the *Betalain* color marker. The F1 somatic recombination event generated twin sectors as seen in **B)**. The putative Chromosome 3 genotypes of each sector are presented in **D)**. In the viable F2 plant with CO event, a gamete containing the Chromosome 3 somatic CO product of the purple sector paired with a gamete containing the Chromosome 3 MT parental type. Gray dot, centromere. Purple arrow, *Betalain* T-DNA integration site. Black lightning bolt, DSB site. Orange highlight, heterozygous M82/MT SNPs; red highlight homozygous M82 SNPs; yellow highlight, homozygous MT SNPs.

To search for twin sectors for the Chromosome 3 target (Fig. 2C), 10 F1 light purple (*Bet/−*) plants containing SpCas9 + *PSY1* gRNA and 10 F1 purple (*Bet/−*) control plants containing SpCas9 but lacking the gRNA (Supplemental Table S3) were grown in the greenhouse. Most plants appeared to be phenotypically heterozygous (light purple) with no twin sectors large enough to represent a high-confidence CO event (Fig. 3A). A flower of 1 of the plants showed, in a petal, a somatic twin sector consisting of a dark purple *Bet/Bet* sector adjacent to a yellow *−/−* (WT) sector in the background of light purple *Bet/−* (Fig. 3B; Supplemental Table S4).

The twin sectors on the petals were large, occupying about half of the petals. Therefore, the event giving rise to this sector must have happened at an early stage of floral development and thus might have been transmitted into the lineage of the gametes. To test this, seeds from the fruit generated from this chimeric flower were extracted and germinated and produced 6 viable F2 progeny plants. Among them, 1 light purple F2 (*Bet/−*) plant had a CO event on Chromosome 3. This was confirmed by genotyping around the DSB: on the telomere proximal side, both the *PSY1* gRNA site and the SNP closest to the *PSY1* gRNA were heterozygous (M82/MT), while the next SNP proximal to the centromere was homozygous MT (Fig. 3, C and D; Supplemental Table S5). This plant was analyzed by whole-genome sequencing (WGS) and did not exhibit changes in the number of reads along both sides of the DSB site (Supplemental Fig. S1). Therefore, this change from heterozygote to homozygote is unlikely to be due to a chromosome segment loss. The plant was fertile and produced viable F3 seeds further confirming that this was a genuine targeted CO. We did not find CO events in 10 F2 progeny of other fruit of the same plant nor in 10 F2 progeny of M82 pX11 × MT SpCas9 control plants lacking *PSY1* gRNA.

For Chromosome 11 (Fig. 2D), where the pX11 cassette was mapped to SL4.0ch11:47,305,369, we found an additional visual marker on the long arm of Chromosome 11: the *CLAVATA3* (*CLV3*) gene located at position SL4.0ch11:52,945,095, between the pX11 cassette and the telomere. Seeds of the *clv3-1* mutant in the M82 background were kindly provided by Zach Lippman's lab. The *clv3* mutants exhibit a fasciated phenotype in flowers and fruits (Xu et al. 2015), seen as an increase in floral organs and fruit locules compared to WT plants. We used *clv3* as an additional genetic marker to detect recombination or other chromosomal rearrangements. *CLV3* is 5.82Mb upstream from gRNA2 proximal to the telomere. The transgenic M82 *clv3* plant with SpCas9 and gRNA2 was crossed with MT pX11. Ten F1 light purple (*Bet/−; CLV3/clv3*) plants containing SpCas9 + gRNA2 and 10 F1 purple (*Bet/−; CLV3/clv3*) control plants containing SpCas9 but lacking the gRNA (Supplemental Table S3) were grown in the greenhouse. After 8 wk, all plants appeared phenotypically heterozygous for the *Bet* marker (light purple) with no visible twin sectors large enough to represent COs and no green sectors expected of LOH events.

### Genotyping and phenotyping of somatic LOH events

**Figure 4.**
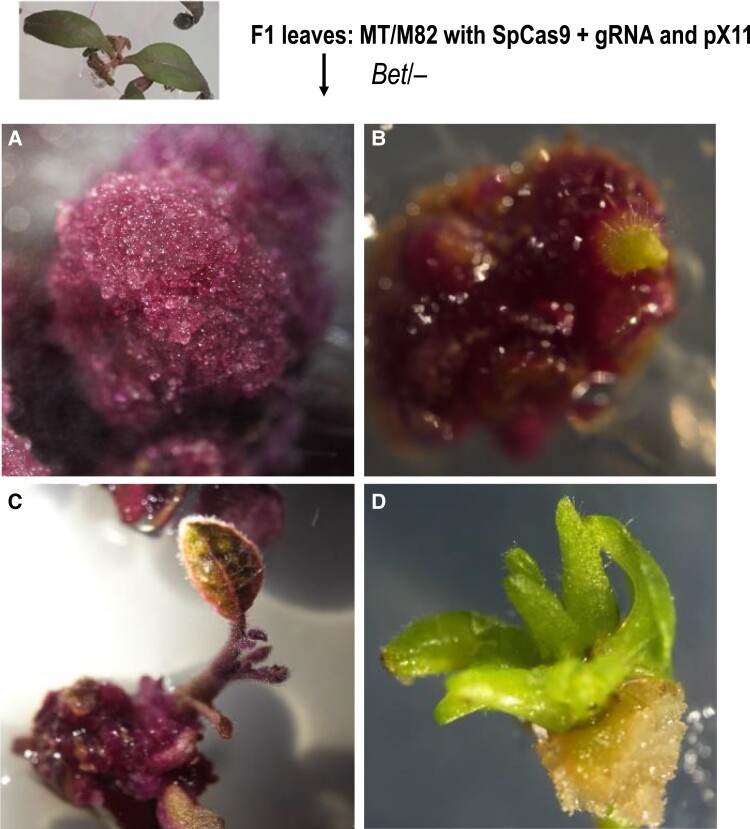
Screening for green sectors regenerated from F1 somatic tissues. We analyzed F1 light purple and SpCas9 positive plants of the genotypes ([MT SpCas9 + *PSY1* gRNA]/[M82 pX11 on Chromosome 3]) or ([M82 *clv3* SpCas9 + gRNA2]/[MT pX11 on Chromosome 11]). Green and light purple plantlets regenerated from pieces of light purple F1 leaves were obtained. A light purple callus **A)** and a light purple plantlet **C)** were regenerated from a light purple F1 leaf. Green calli **B)** occasionally emerged from a purple callus giving rise to a green regenerated plantlet **D)**. Green plantlets were further analyzed as potential LOH products.

As the presence of large sectors is a rare occurrence, we wanted to have an additional screen that allows the detection and regeneration of whole plants from small sectors. To do it, we grew 10 F1 seedlings each for Chromosomes 3 and 11 markers in sterile conditions for regeneration from cotyledons to search for small green somatic sectors. Our plan was to regenerate whole plants from these sectors and identify the underlying cause of their phenotypes. We hypothesized that the F1 plants either experienced LOH or that the transgene was silenced. Two-wk-old purple seedlings (*Bet/−*), confirmed for SpCas9 presence (Supplemental Table S3), were dissected into small pieces and transferred into tissue culture for whole-plant regeneration. On average 160 to 180 leaf pieces per plant were prepared from each of the 10 F1 purple (*Bet/−*) plants. Calli and newly emerging plantlets were identified, and green plantlets were observed (Fig. 4).

For each green regenerated plant, the presence/absence of the pX11 T-DNA, or at least 1 of its border junctions with the genomic integration site, was verified by PCR. For Chromosome 3, 2 F1 green plants were regenerated, representing candidates for LOH. We found that the plants did not lose the pX11 T-DNA; thus we concluded they were silencing events. One silencing event was also observed among the control F1 purple (*Bet/−*) SpCas9 plants. For Chromosome 11, 1 silencing event was observed among F1 purple (*Bet/−*; *CLV3/clv3*) SpCas9 + gRNA2 plants and 1 in the control among F1 purple (*Bet/−*; *CLV3/clv3*) SpCas9 plants. These data suggest that gene silencing was not related to the occurrence of distant DSBs. Thus, these silencing events (Supplemental Table S4) were not further analyzed.

**Figure 5.**
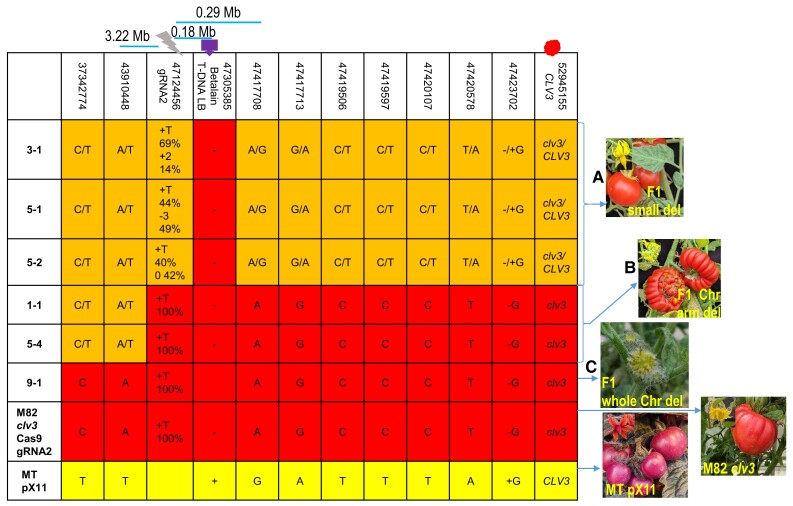
SNP genotyping of regenerated F1 green plant. Six regenerated green plantlets derived from F1 hybrids and their parents were sequenced for SNP markers in the region around the Chromosome 11 gRNA2-induced DSB and for the *CLV3* marker genotype and phenotype. Coordinates of SNPs on SL4.0 Chromosome 11 are noted in the top row of the table. The DSB point is marked as a gray lightning bolt. **A)** In Plants 3-1, 5-1, and 5-2, the T-DNA was missing. SNPs were heterozygous for the genotype of both parents, upstream and downstream of the DSBs all the way to the *CLV3* gene, which is located ∼2 Mb distal from the telomere, as expected for an F1. Flowers and fruit had a *CLV3* WT phenotype. **B)** In Plants 1-1 and 5-4, the T-DNA was missing. Upstream of the DSB point, SNPs were heterozygous. After the DSB and all the way to the *CLV3* gene, SNPs were homozygous M82. Flowers and fruit had a *clv3* fasciated phenotype. **C)** In Plant 9-1, the T-DNA was missing. Upstream and downstream of the DSB and SNPs, including the *CLV3* gene, were homozygous for M82. Flowers had an extreme *clv3* fasciated phenotype, and no fruits were generated. Orange highlight, heterozygous M82/MT SNPs; red highlight, homozygous M82 SNPs; yellow highlight, homozygous MT SNPs. Chr, chromosome; del, deletion. 3.22 Mb, distance between gRNA2 and the first SNP downstream proximal to the centromere. 0.18 Mb, distance between the *Betalain* T-DNA integration site and gRNA2. 0.29 Mb, distance between the first SNP upstream proximal to the telomere and gRNA2. Gray lightning bolt, DSB site. Purple arrow, Betalain T-DNA integration site. Red dot, *CLV*3 position.

In addition to the plants carrying gene silencing events, other green regenerated plants were analyzed (Fig. 5; Supplemental Table S4). Three such plants (Fig. 5, Plants 3-1, 5-1, and 5-2) had lost the T-DNA insertion region but were heterozygous for all SNPs around the T-DNA, including in the region of the gRNA target, as expected for F1 plants (Fig. 5; Supplemental Table S5). Moreover, these plants also had the expected F1 *CLV3* genotype (*CLV3/clv3*) and phenotype (Fig. 5A) and were fertile, suggesting that no major chromosomal rearrangements had occurred.

**Figure 6.**
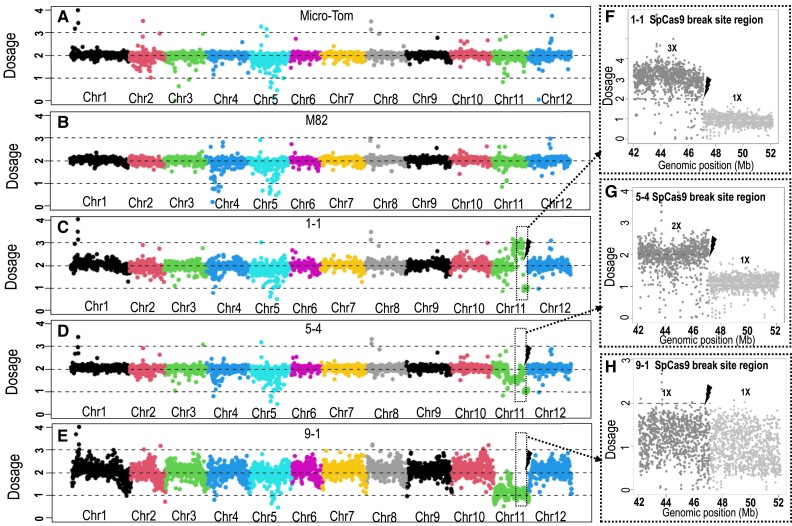
Dosage changes at the induced DSB site by WGS coverage analyses. Average coverage of WGS reads per plant was determined as 2× diploid dosage basis. **A)** to **E)** Coverage for each of the 12 chromosomes per plant is presented with each chromosome shown in a different color. **A)** and **B)**: parental plants with 2× in all chromosomes. **C)**: Plant 1-1 showing deviation of 2× ploidy in Chromosome 11. **F)** Plant 1-1 zoom-in on Chromosome 11, about 5 Mb from each side of the gRNA2 DSB site. The dosage in each region reveals changes in ploidy levels. Plant 1-1 shows transition at the DSB site, from a dosage of 3× to 1× (likelihood ratio test *P* < 0.001). This indicates loss of the region from the DSB site to the telomere in 1 of the chromosomes. **D)** Plant 5-4 showing deviation of 2× ploidy in Chromosome 11. **G)** Plant 5-4 zoom-in on Chromosome 11, about 5 Mb from each side of the gRNA2 DSB site. The dosage in each region reveals changes in ploidy levels. Plant 5-4 shows transition at the DSB site, from a dosage of 2× to 1× (likelihood ratio test *P* < 0.001). This indicates loss of the region from the DSB site to the telomere in 1 of the chromosomes. **E)** Plant 9-1 shows a dosage of 2× along all chromosomes, but throughout Chromosome 11, there is a dosage of 1× indicating loss of the whole chromosome from 1 of the parents. **H)** Plant 9-1 zoom-in on Chromosome 11, about 5 Mb from each side of the gRNA2 DSB site. The dosage in each region reveals no change in the ploidy levels showing a dosage of 1× on both sides of the DSB site (likelihood ratio test *P* > 0.05). Chr, chromosome. Black lightning bolt, DSB site.

Two plants lacked the T-DNA pX11 cassette and showed a transition from heterozygous SNPs to SNPs homozygous for the M82 parent (Fig. 5, Plants 1-1 and 5-4) in the DSB region. The plants were also homozygous for the telomeric *clv3* allele mutation and had severely fasciated flowers and fruits (Fig. 5B). These plants could have been considered to exhibit targeted CO events, based on their SNP genotype and the phenotype. However, these phenotypes and genotypes could also be explained by a loss of chromosome arm from the DSB site to the telomere. This possibility was strengthened by the high sterility of the plants and was further confirmed through WGS (see below) (Fig. 6).

Plant 9-1 also lacked the T-DNA pX11 cassette and was homozygous for all the M82 SNPs. Moreover, it was homozygous for the *clv3* allele, it had the most severely fasciated flower phenotype, and it was totally sterile, bearing no fruit (Fig. 5C). This genotype/phenotype could be explained by a complete chromosomal loss, as confirmed by WGS analysis described below (Fig. 6).

### WGS analysis of LOH events

WGS was performed for Plants 3-1, 5-1, 5-2, 1-1, 5-4, and 9-1 and for the MT and M82 lines. The sequencing coverage in Plants 3-1, 5-1, and 5-2 (Supplemental Fig. S2, A to C, respectively) was similar to that of the parental plants, MT and M82 (Fig. 6, A and B). The ploidy dosage was determined using sequencing coverage analysis as described in the Materials and Methods. SNPs in the reads could be anchored to the parental sequences, and therefore this analysis enabled assessing deviations from the diploid dosage as well as homozygosity, heterozygosity, and hemizygosity.

Analysis of F2 progeny of Plant 5-1 homozygous for MT SNPs on both sides of gRNA2 and pX11 that revealed a 4069-bp fragment between the NOS terminator inverted repeats was missing from the pX11 T-DNA on Chromosome 11 (Supplemental Fig. S3A). Except for the deleted part of the pX11 T-DNA in Plant 5-1, the dosage was 2× in all chromosomes, including Chromosome 11.

WGS of F2 progeny of Plants 3-1 and 5-2, which were also homozygous for MT SNPs on both sides of gRNA2 and pX11, showed that a 17,396-bp fragment of Chromosome 11 (SL4.0ch11:47,301,154-47,318,550) spanning the original T-DNA insertion site (SL4.0ch11:47,305,369) was missing, as confirmed by Sanger sequencing (Fig. 5). Except for the deleted parts spanning the pX11 T-DNA in Plants 5-1 and 5-2, the dosage was 2× in all chromosomes, including Chromosome 11. Both sides of the deleted region spanning the T-DNA are flanked by A-rich repeats (Supplemental Fig. S3B). Note that T-DNA loss was not detected in control plants where no DSB induction occurred.

WGS of F1 9-1 showed the expected 2× coverage and SNP heterozygosity throughout the genome except for Chromosome 11 where significant deviations were observed (Fig. 6E). The coverage dosage was 1× throughout Chromosome 11, and SNPs corresponded only to the M82 parent (Fig. 6E). Note that such massive loss was detected only in plants containing Cas9 and gRNA where a DSB was induced, and not in control plants.

WGS of Plants 1-1 and 5-4 confirmed the transition from heterozygous to M82 SNPs precisely at the SpCas9-induced DSB site (Fig. 5). The analysis of dosage of sequencing reads showed that in both 1-1 and 5-4, the dosage around the targeted DSB site changed from 3× (possible duplication) or 2× (diploid), respectively, to 1× (haploid). This result indicates that a segment from the DSB region proximal to the telomere of the long arm of Chromosome 11 was lost (Fig. 6, C, D, F, and G) as it would be expected for unrepaired DSB events.

WGS of F1 9-1 showed the expected 2× coverage and SNP heterozygosity throughout the genome except for Chromosome 11 where significant deviations were observed (Fig. 6E). The coverage dosage was 1× throughout Chromosome 11, and SNPs corresponded only to the M82 parent (Fig. 6, E and H), with the exception of reads across the centromere, possibly due to mapping biases. Note that such massive loss was detected only in plants containing Cas9 and gRNA where a DSB was induced, and not in control plants.

### Multiple chromosomal rearrangements are associated with telomere loss

**Figure 7.**
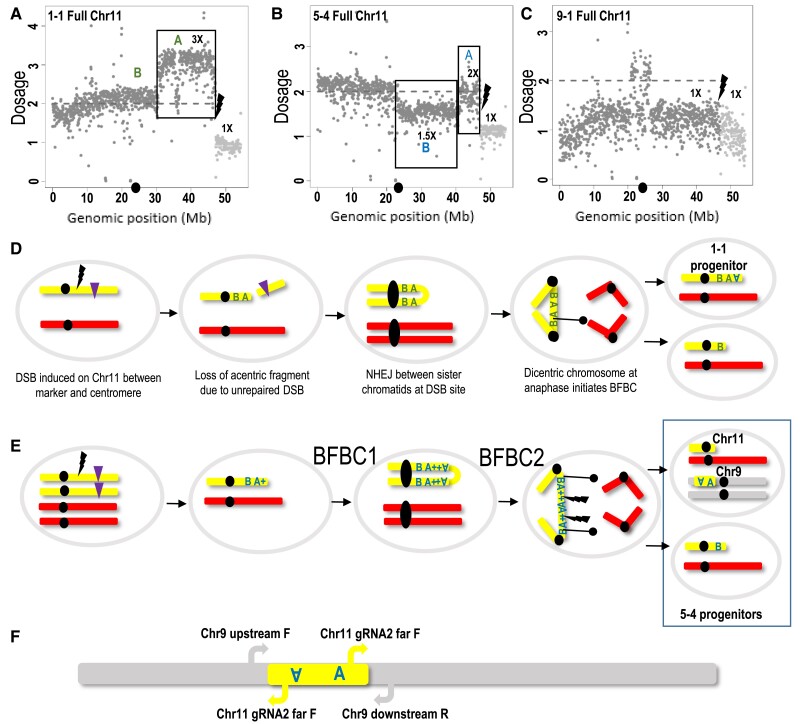
Analysis of chromosomal rearrangements at DSB sites and an underlying mechanistic model of the BFBC and chromothripsis. **A)** Plant 1-1 whole Chromosome 11 coverage shows transition at the DSB site, from a dosage of 3× to 1×. This indicates loss of the region from the DSB site to the telomere in 1 of the chromosomes and duplication in the other side of the DSB marked by a gray A. **B)** Plant 5-4 whole Chromosome 11 coverage reveals changes in ploidy levels, which are more complicated compared to Plant 1-1. Plant 5-4 shows transition at the DSB site, from a dosage of 2× in the region marked by blue A to 1× from the DSB site to the telomere. This indicates loss of the region from the DSB site to the telomere in 1 of the chromosomes. An additional change from the average coverage is seen in the region marked by a blue B, which has a dosage of 1.5×. **C)** Plant 9-1 whole Chromosome 11 coverage. In this plant, the dosage is 1× throughout the whole Chromosome 11, except in the centromere region. **D)** Illustration of a putative BFBC that could explain the 3× dosage of the region marked by a gray A in Plant 1-1. **E)** Illustration of putative 2 rounds of BFBC that could explain the 2× dosage of the region marked by a blue A and the 1.5× dosage of the region marked by blue B in Plant 5-4. The first round is similar to Panel **D)** illustration. But here maybe the DSB occurred in the stage where 2 chromatids are present. The broken chromatid may have invaded the intact chromatid and copied the region containing the gRNA2 target site marked by +. In the second round, the bicentric chromosome may have broken at several points. A duplicated part of the region marked by blue A+ that contains the inverted duplicated A region and the gRNA2 target site from each side of the duplication may have been cut by the SpCas9 and translocated into Chromosome 9. **F)** Scheme of the Chromosome 11 segment translocated into Chromosome 9. We used 2 PCR primer sets to amplify and sequence the junctions. Chr9 upstream F + Chr11 gRNA2 far F for the upstream junction. The amplification of this junction using the Chr11 gRNA2 far F primer indicates the inverted orientation of the Chromosome 11 blue A region. Chr11 gRNA2 far F and Chr9 downstream R used to amplify the downstream junction indicates the forward orientation of the Chromosome 11 blue A region. MT Chromosome 11 is highlighted in yellow; M82 Chromosome 11 in red; Chromosome 9 in gray. Black lines with a black circle indicate a random DSB site generated by pulling of the bridge part to 2 opposite poles in the dicentric chromosome. The black lightning bolt indicates the SpCas9 DSB site. The Chromosome 11 centromere is at 23.24 Mb and is marked by a black dot on the genomic position (Mb) axis.

The WGS dosage analysis revealed additional rearrangements in Plants 1-1 and 5-4. Plant 1-1 had a ∼20-Mb region between the DSB and the centromere that became duplicated, showing a 3× chromosome dosage (Fig. 7). This duplication transitioned to chromosome segment showing a 1× dosage precisely at the DSB site (Fig. 7A). This kind of duplication and deletion event could be explained by loss of an acentric chromosome fragment distal to the DSB (and hence the 1× dosage) followed by fusion of 2 centromere-bearing sister chromatids, which would initiate a BFBC (Fig. 7D). During a BFBC, when the 2 centromeres are pulled to the opposite poles in anaphase, a new break is generated randomly along the bridge, eventually leading to a duplication, hence the 3× dosage shown as region A (Fig. 7, A and D) in an inverted orientation (Fig. 7D).

In Plant 5-4, a region between the DSB and the centromere was missing (Fig. 7B). The estimated dosage in this region was 1.5×, which may indicate chimerism. To better understand this event, we examined the DSB junctions using inverse PCR amplifying sequences adjacent to the Chromosome 11 gRNA2 region (Supplemental Table S6). This approach was successful in Plant 5-4, but not in Plant 1-1. Surprisingly, the amplified product showed a junction between the Chromosome 11 gRNA2 region and regions located on Chromosome 9 (Fig. 7F). We then designed a primer to the putative Chromosome 9 sequence and verified the junction by PCR and Sanger sequencing (Supplemental Table S7 and Fig. S4A). We also verified the upstream junction of the Chromosome 11 gRNA region using a primer targeting the putative upstream Chromosome 9 sequence and the primer from the Chromosome 11 gRNA2 region (Figs. 7F and S4B). The translocation represented by the 2 junctions was further confirmed by the finding of WGS Illumina reads corresponding to the chimeric junctions between Chromosomes 11 and 9 (Supplemental Fig. S5). The directionality of the joined Illumina reads and the primers used for PCR amplification of the junctions support the model shown in Fig. 7E, suggesting that in 5-4, the A inverted repeat region was broken and translocated to Chromosome 9 (Figs. 7, E and F, and S4 and S5). Such rearrangement is similar to events resulting from chromothripsis (Stephens et al. 2011; Ostapińska et al. 2022). We did not find any potential SpCas9-gRNA2 target sites in the Chromosome 9 translocation region that could explain why the A fragment was inserted specifically at that location. Taken together, the complex rearrangements in Plant 5-4 might be best explained through a scenario where the first BFBC round, which was similar to that in Plant 1-1 (Fig. 7D), was followed by a second round in which the A duplicated region was broken and translocated to Chromosome 9. The second daughter cell would contain the broken MT Chromosome 11 with the blue B region. These 2 daughter cells generated after the second BFBC may be the progenitors of the tissues analyzed in Plant 5-4 (Fig. 7E).

In Plant 9-1, the coverage dosage was 1× throughout Chromosome 11, with SNPs corresponding only to the M82 parent (Fig. 7C), except for the centromere region (23.24 Mb), where the dosage was 2×. Since the centromere region is rich in repeated sequences, it is difficult to determine if this is a misalignment artifact and in fact the whole MT chromosome was lost. Alternatively, it might be a near-complete loss of Chromosome 11, with only a minichromosome consisting of the Chromosome 11 centromere remaining.

**Figure 8.**
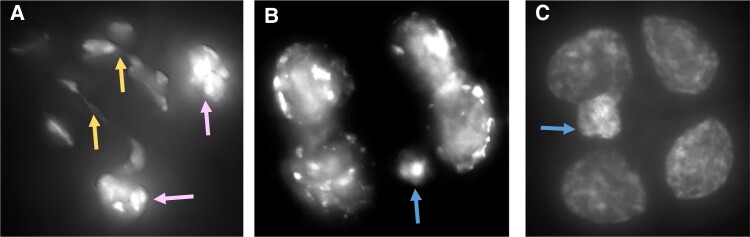
Cytological evidence of chromosomal rearrangements and micronuclei in meiocytes. **A)** Plant 1-1 meiocyte at late anaphase I showing 2 chromosome bridges (yellow arrows) and 2 clumps of chromosomes that have not properly segregated to daughter cells (pink arrows). **B)** Plant 1-1 tetrad with a micronucleus (blue arrow). **C)** Plant 5-4 tetrad with a micronucleus (blue arrow). Meiocytes from young flower buds were fixed in 3:1 ethanol:acetic acid and stained with DAPI.

Cytological analysis of Plants 1-1 and 5-4 pollen mother cells revealed the presence of bridges during meiotic anaphase I (Fig. 8A) and micronuclei in tetrads (Fig. 8, B and C). The detection of bridges further suggests the presence of the chromosomal rearrangements detected by WGS. The rearrangements could be the results of prior BFBC episodes. The presence of micronuclei is also consistent with either the presence of acentric chromosome fragments or chromosomes that cannot pair properly, presumably as the result of their rearrangements.

## Discussion

We have developed a system that enables visual identification of a wide range of chromosome alterations. We report examples of such alterations, including a CO and LOH due to chromosomal rearrangements ranging from segment to whole chromosome loss or translocation. The system is based on a previously mapped visual transgenic marker. In principle, the *Betalain* marker can serve to monitor LOH on any chromosome as well as in many plant species, making this system quite general. This marker can be combined with additional phenotypic markers, as done here with the recessive *clv3* mutation on Chromosome 11, to facilitate the analysis of the studied events.

We showed that twin sectors could be useful in detecting a targeted CO at the site of induced DSBs located between the centromere and the marker on Chromosome 3 (Fig. 3). We found a single large twin sector in a flower of 1 out of 10 plants, and we confirmed in the progeny of this flower that a germinally transmitted targeted CO took place at the DSB induction site. Such CO could give rise to reciprocal events, either a purple (i.e. containing the T-DNA marker) or a green WT transgene-free targeted recombinant. In this single event, we obtained a purple plant, but in principle, the system can be used also for the recovery of transgene-free germinal recombinant events making it a useful tool. The potential benefits of such systems in plant breeding have been thoroughly discussed (Taagen et al. 2020; Sukegawa et al. 2021). The rate of occurrence of twin sectors was low, which is consistent with our previous works on targeted somatic COs in tomato (Filler-Hayut et al. 2017; Ben Shlush et al. 2020) and *Arabidopsis* (Filler-Hayut et al. 2021). Presumably, low somatic CO rates might be due to a lack of machinery for homolog pairing and for double-Holliday junction formation and resolution as found in meiocytes. However, the system described here is not restricted to a particular chromosomal site and can be applied to screen for rare CO events thanks to the visual marker, making it useful for plant breeding, adding a new tool to the chromosome engineering panoply (Rönspies et al. 2021). In addition, it can be used to screen for regions in the genome, or for mutants, that are more prone to repair via HR and via CO. Before, we used a natural marker at the *Sulfur* locus in tobacco (*Nicotiana tabacum*) to identify the *Hyrec* mutant exhibiting high somatic recombination rates (Gorbunova et al. 2000). The *Hyrec* locus has not been characterized at the molecular level, but its phenotype shows that reaching high rates of somatic CO might be feasible.

Because of the low number of large green or twin sectors seen in whole plants and to enable obtaining of germinal from somatic events, we also screened for green calli regenerated from leaves of light purple F1 plants. Such events could be preexisting in the leaves, becoming visible through the regeneration process. Alternatively, they could be stimulated by the regeneration process: the repair process of the CRISPR-induced break might be less efficient in rapidly cycling tissue culture cells than during normal plant development. This effect, however, would be locus-dependent as, when testing the Chromosome 3 euchromatic target, we did not detect WT sectors corresponding to DSB-induced LOH. With the heterochromatic Chromosome 11 DSB target, we found 6 independent LOH events. All of these events were deletion events of various sizes and not CO events. The abundance of repeats in the Chromosome 11 DSB target might have triggered defective repair as previously reported for centromeric regions (Barra and Fachinetti 2018). These LOH events were genotyped locally around the break and genome-wide, using WGS. In 3 out of these 6 plants, the *Betalain* T-DNA was fully or partially eliminated. The mechanistic underpinnings of these transgene elimination, 180 kb away from the DSB, events are not clear. It is well known that somaclonal variation induced in tissue culture can give rise to chromosomal rearrangements, even without DSB induction. However, we did not detect T-DNA loss in SpCas9 control plants. It is possible that the deletions were induced through a long-range repair mechanism that remains to be discovered. Note that off-site DSBs are an unlikely explanation for T-DNA loss as deletions occurred in regions with no homology to the target, namely, the NOS inverted repeats and an A-rich region.

Regarding the Chromosome 11 loss in Plant 9-1, it is conceivable that a DSB-induced BFBC could lead to a very small and unstable chromosome, and this is further supported by the lack of similar loss in control plants that underwent the same tissue culture process but no DSB induction. Here too, we do not have direct evidence connecting the CRISPR-Cas-induced DSB and the chromosomal loss, except not finding such events in SpCas9 control plants. However, at least in 2 cases (Plants 1-1 and 5-4), the deletions took place precisely at the DSB site and did not occur in controls, suggesting that these are bona fide DSB-induced events rather than being tissue culture-induced. The frequency of such genomic loss events was very low. Out of 1,600 to 1,800 regenerated leaf pieces and several calli per leaf, we obtained 3 events of small deletions, 1 event of whole chromosome loss, and 2 events of chromosome arm loss from the DSB site to the telomere. Moreover, the 3 loss events of larger chromosomal sequences generated sterile F1 plants.

The analyses of Plants 1-1 and 5-4, including genotyping around the break, phenotypic marker analysis, WGS, and cytological analyses, supported the hypothesis of the presence of large chromosomal rearrangements, consistent with BFBC (McClintock 1941). In plants, BFBC was characterized by McClintock several decades ago (McClintock 1941). Interestingly, McClintock's BFBC turned out to be induced by a double-Ds transposon, which might have generated DSBs more challenging for the cell to repair than those made by simple Ac/Ds elements which generate simple excision footprints rather than large-scale chromosomal rearrangements (English et al. 1993; Weil and Wessler 1993). Likewise, in this work, 1 of the loci did not trigger BFBC, while the other (on Chromosome 11 in a heterochromatic region) did. CRISPR-Cas-induced BFBC has not yet been reported in plants and understanding which loci are repaired in a “clean” manner and which ones undergo a defective repair that generates large-scale genomic rearrangements is very limited.

In mammalian cells, the phenomenon of CRISPR-Cas-induced BFBC has been recently reported, and it is an important concern for the field of gene therapy (Zuccaro et al. 2020; Alanis-Lobato et al. 2021; Leibowitz et al. 2021). There, it is associated with a series of massive rearrangements, including micronuclei formation and translocation of chromosomal segments into new chromosomal locations—a phenomenon called chromothripsis (Kwon et al. 2020; Ostapińska et al. 2022). We report here on a similar syndrome of catastrophic chromosomal rearrangements, namely, the occurrence of BFBC, micronuclei, chromosome loss, and translocations, showing that CRISPR-Cas-induced breaks can trigger chromothripsis-like phenomena in plants. Unlike with gene therapy in humans, the phenomenon of CRISPR-induced chromothripsis is of no concern in plant breeding, due to its rarity. Moreover, the major chromosomal rearrangements it induces would be deleterious and most probably would cause sterility. The genetic system developed here, with easy-to-monitor phenotypic markers, paves the way to study the phenomenon of DSB-induced chromothripsis in plants.

## Materials and methods

### Plant material

Tomato (*S. lycopersicum*) M82 *clv3-1* seeds were a kind gift from the Zachary B Lippman’s Lab (Xu et al. 2015). MT pX11 seeds were a kind gift from the Asaph Aharoni lab (Polturak et al. 2017). Tomato plants were grown in a greenhouse in 5-L pots with controlled climate conditions of 26 ± 1 °C and a light period of up to 14 h. The light source is sunlight supplemented by full-spectrum light bulbs. The daily light integral is about 27 to 30 moles/m^2^/d.

### Plasmids

DSB induction was performed by *Streptococcus pyogenes* Cas9 (SpCas9). *Arabidopsis* (*A. thaliana*) optimized SpCas9 was expressed under the parsley (*Petroselinum crispum*) ubiquitin promoter, Ubi4-2, and pea (*Pisum sativum*) 3A terminator (Fauser et al. 2014). The gRNAs were expressed under the *Arabidopsis* U6-26 promoter. A kanamycin resistance gene was expressed under the *Nopaline Synthase* (Nos) promoter and Nos terminator (referred to as Nos:NptII:Nos). Plasmids were cloned using the GoldenBraid system (Sarrion-Perdigones et al. 2014).

### Plant transformation

Transformation of tomato plants was done using *Agrobacterium tumefaciens* containing our cloned plasmid. M82 cotyledon transformation was done according to a protocol previously described (Dahan-Meir et al. 2018).

### Genomic DNA extraction

Two or 3 small tomato leaflets were collected into 1.5-mL tubes and ground. Extraction was done according to a protocol previously described (Dahan-Meir et al. 2018), except for genomic DNA for WGS that was extracted using NucleoSpin DNA purification kit (MACHEREY-NAGEL).

### DNA amplification and sequencing

CRISPR-Cas9 footprints of the parent MT SpCas9 + *PSY1* gRNA plant were analyzed using HTS of PCR amplicons (Dahan-Meir et al. 2018). CRISPR-Cas9 DSB regions of the F1 plants ([MT SpCas9 + *PSY1* gRNA]/[M82 pX11 on Chromosome 3]), parent M82 *clv3* SpCas9 + gRNA2 plant 2, and F1 plants ([M82 *clv3* SpCas9 + gRNA2]/[MT pX11 on Chromosome 11]) were PCR amplified and Sanger-sequenced. We used the Tracking of Indels by DEcomposition (TIDE) web tool for analysis of the Sanger sequencing CRISPR-Cas9 footprints (Brinkman et al. 2014).

### Inverse PCR

An inverse PCR protocol was used to determine the location of pX11 (Betalain) T-DNA in the M82 and MT lines. Nested primers were designed and used in the inverse PCR reaction (Thomas et al. 1994) (Supplemental Table S1). Genomic DNA (300 ng) from leaves of M82 and MT pX11 lines was incubated at 37 °C overnight with PstI-HF or HindIII-HF (New England BioLabs) restriction enzymes, respectively, followed by 20 min at 65 °C for restriction enzymes inactivation. Then, 150 ng of the digested fragments were self-ligated with T4 DNA ligase (New England BioLabs) for 2 h at room temperature. The self-ligation reaction was used for the first and second PCR reactions with nested primers, between which the PCR products were cleaned using magnetic beads. The second PCR products were cleaned and sequenced by Sanger sequencing to identify the genomic region flanking the T-DNA LB. Based on the putative genomic integration site, primers were designed for amplification and sequencing of the genomic sequences and the LB or RB junctions. An inverse PCR protocol was also used to detect the translocation of Chromosome 11 sequence into Chromosome 9 in the 5-4 green F1 plant. Genomic DNA was digested with PstI restriction enzyme. Nested primers were designed and used in the inverse PCR reaction (Thomas et al. 1994) (Supplemental Table S6). The rest of the protocol is as above. We sequenced the PCR product by Sanger sequencing to identify the genomic region of the translocation downstream junction. Additional PCR primers were designed for verification and Sanger sequencing of Chromosome 11 into Chromosome 9 translocation junctions (Supplemental Table S7)

### Plant regeneration

F1 plants were sterilized and sown in boxes containing Nitsch growth medium, and once grown, their leaves were dissected to small ∼0.5-cm^2^ pieces. They were then transferred to the selection medium I, as described in the plant transformation section above, but without kanamycin. The leaves were then gradually transferred to plates containing selection mediums II and III and then to boxes with rooting medium and to 5-L pots in the greenhouse.

### SpCas9 + gRNA transgenic plant generation

The gRNAs were cloned into a GoldenBraid vector (Sarrion-Perdigones et al. 2014). *PSY1* gRNA was cloned into p3Ω1 nos:nptII:nos ubi:SpCas9 U626:*PSY1* gRNA. gRNA2 was cloned into p3Ω1 nos:nptII:nos ubi:SpCas9 U626:gRNA2.

The MT SpCas9 + *PSY1* gRNA plant #10 was previously selected (Dahan-Meir et al. 2018) and was used for crosses with M82 pX11 plants. The heterozygous (50% −GCT/50% −G) footprint in the gRNA recognition sequence in this plant created the conditions for an allele-specific assay, in which a DSB will occur only in the M82 parent (Supplemental Table S2).

M82 *clv3* cotyledons were transformed with the gRNA2 cassette, regenerated rooted plants were transferred successfully to the greenhouse, and transformant plant tissue was collected for DNA extraction and sent to Sanger sequencing of the target area. The presence of SpCas9 was confirmed in all plants through PCR amplification with primers from within the Cas9 sequence (Supplemental Table S3). Different NHEJ repair footprints were observed, and 1 specific line (M82 *clv3* SpCas9 + gRNA2 plant #2) was chosen for crosses with MT pX11 plants. The homozygous (100% +T) footprint in the gRNA recognition sequence in this plant created the conditions for an allele-specific assay, in which a DSB will occur only in the MT parent (Supplemental Table S2).

### SNP analysis by Sanger sequencing

To analyze SNPs between M82 and MT at the DSB target sites and from both sides of the Chromosome 3 *PSY1* gRNA or Chromosome 11 gRNA2 targets, we performed PCRs of each selected SNP region, on genomic DNA. The PCR products were then sequenced by the Sanger method. The details of the PCR primers and their genomic location are in Supplemental Table S5.

### WGS and analysis

DNA was purified from leaves of F1 and F2 plants using a DNA purification kit (MACHEREY-NAGEL), and then 300 ng was sheared by sonication (Bioruptor, Diagenode) to 200 to 500 bp. A total of 10 ng of fragmented DNA per plant was used for library preparation as described in (Ben Shlush et al. 2020). High-throughput sequencing was performed at the Life Sciences Core facilities unit at the Weizmann Institute of Science with the Illumina NovaSeq 6000, 150-bp paired-end reads. The coverage of the various genomes was ×5 to ×31 (Supplemental Table S8). The WGS reads of the selected tomato plants were aligned to the SL4.0 tomato genome (Sol Genomic Network), as in (Ben Shlush et al. 2020). The reads were viewed and further analyzed using the IGV browser (Robinson et al. 2011).

### Coverage analysis

Average coverage was computed using GATK version 3.7. The genome was divided into windows of 250 kb for genome-wide analyses and 5 kb for more detailed inspections of the expected DNA double-strand break site. Dosage was estimated by normalizing the observed number of reads per window by the mean number of reads, stratified by GC content to account for differences in coverage due to GC content bias. GC content was computed with bedtools nuc version 2.26 across windows. Windows were then subdivided in deciles of GC content, and the mean number of reads was estimated for each decile and then used for normalization. The presence of chromosomal rearrangements on each side of the Cas9 target site was tested comparing with a likelihood ratio test models in which no rearrangements, a full deletion of both chromosomes, a single chromosomal arm deletion or a duplication, occurred after the break assuming a dosage of 2×, 0.01×, 1×, or 3×, respectively, and considering 1 Mbp before and after the break. Rearrangements supported by a *P*-value lower than 0.001 were considered as true rearrangements. The R code used for plotting and testing of the rearrangements can be found at github.com/fabrimafe/CRISPRcoverage.

### Imaging of meiocytes

Tomato flower buds were fixed in ethanol:acetic acid 3:1 for at least 1 h and then kept in 70% ethanol at 4 °C. Anthers of flower buds were dissected and stained in 50 ng/*µ*L DAPI in ProLong Gold Antifade Mountant. For imaging, we used a Nikon Eclipse Ti microscope and a 100× Nikon N plan Apo lambda 100x/1.45 oil OFN25 DIC N2 lens. Images were transferred into the DeltaVision (Applied Precision) format, deconvolved, and examined in 3D. The images presented in Fig. 8 are flat projections of the 3D images.

## Supplementary Material

koad209_Supplementary_Data

## Data Availability

Data from WGS have been deposited in NCBI under the BioProject identifier PRJNA991125 and Sequence Read Archive SRP447285.

## Supplemental data

The following materials are available in the online version of this article.

**Supplemental Figure S1.** WGS coverage analyses show no dosage changes at the induced DSB site of Chromosome 3 CO event.

**Supplemental Figure S2.** WGS coverage analyses show no dosage changes at the induced DSB site of Chromosome 11 T-DNA loss events.

**Supplemental Figure S3.** Loss of Betalain expression cassette T-DNA (pX11) in plants 5-1, 3-1, and 5-2.

**Supplemental Figure S4.** PCR amplification of Chromosome 11 translocation into Chromosome 9 junctions from Plant 5-4.

**Supplemental Figure S5.** Illumina reads of Chromosome 11 translocation into Chromosome 9 junctions.

**Supplemental Table S1.** LB primers for inverse PCR.

**Supplemental Table S2.** Cas9 DSB targets on Chromosome 3 and Chromosome 11.

**Supplemental Table S3.** PCR primers used for SpCas9 positive plants screening and selection.

**Supplemental Table S4.** Silencing and LOH events frequencies.

**Supplemental Table S5.** Primers used for sequencing of SNPs in F1, F2, and F3 plants.

**Supplemental Table S6.** Primers used for inverse PCR and detection of Plant 5-4 Chromosome 11 into Chromosome 9 translocation junctions.

**Supplemental Table S7.** Primers used for sequencing of Plant 5-4 Chromosome 11 into Chromosome 9 translocation junctions.

**Supplemental Table S8.** WGS coverage.
