# Supplementary material for: CRISPR/Cas9-induced DNA breaks trigger crossover, chromosomal loss, and chromothripsis-like rearrangements
Source: Plant Cell. 2023 Jul 27;35(11):3957–72. doi: 10.1093/plcell/koad209 (PMC10615209; doi:10.1093/plcell/koad209)
Supplement: koad209_Supplementary_Data [file koad209_supplementary_data.zip › koad209_Supplementary_Data.pdf]

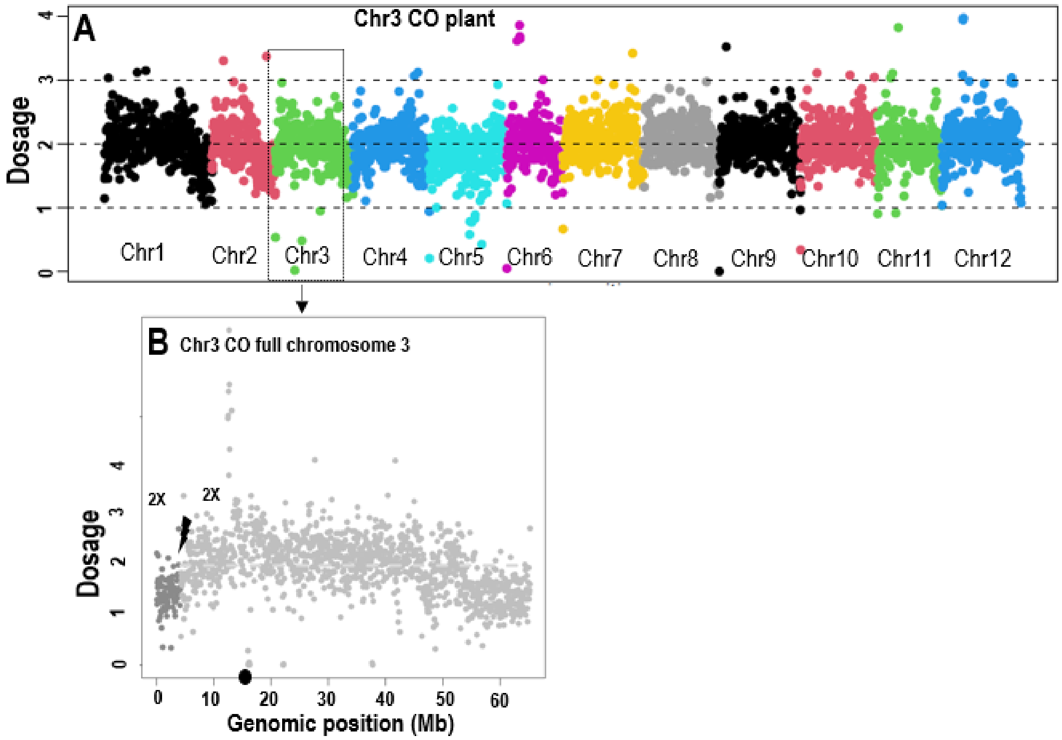

Supplemental Figure S1. WGS Coverage analyses show no dosage changes at the induced DSB site of chromosome 3 CO event.

Supports Figure 3.

Average coverage of WGS reads per plant was determined as 2X diploid dosage basis. A: Dosage for each of the 12 chromosomes, is presented with each chromosome shown in a different color. B: Dark grey dots are dosage bins from genomic position 1 up to the DSB site, and light grey dots are dosage bins from the DSB site to the end of chromosome 3. Chromosome 3 CO plant whole chromosome 3 dosage shows similar ~2X dosage in both sides of the DSB site. This indicates that the genotype transition from both sides of the DSB is not due to loss of the region from the DSB site to the telomere in one of the chromosomes. Chr- chromosome. Black lightning bolt – DSB site. Black dot on Genomic position (Mb) axis – centromere.

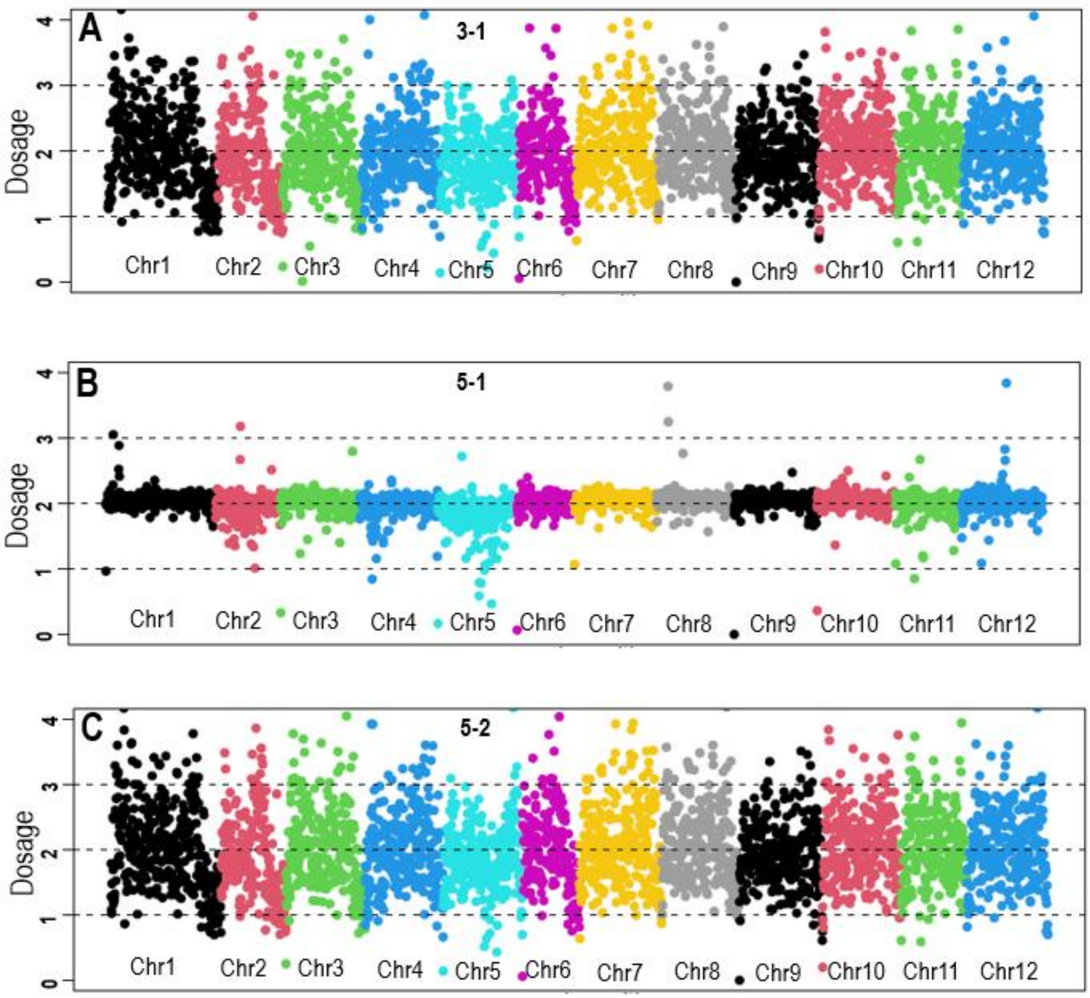

Supplemental Figure S2. WGS Coverage analyses show no dosage changes at the induced DSB site of chromosome 11 T-DNA loss events. Supports Figure 5 and Figure 6.

Average coverage of WGS reads per plant was determined as 2X diploid dosage basis. A-C: Coverage for each of the 12 chromosomes per plant is presented with each chromosome shown in a different color. Panel A: plant 3-1. Panel B: plant 5-1. Panel C: plant 5-2. The dosage in these three plants is 2X in all chromosomes. Chr- chromosome.

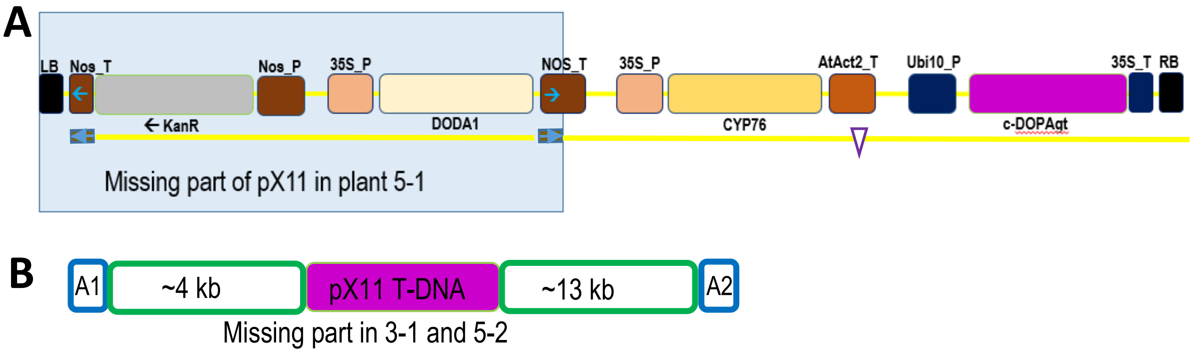

Supplemental Figure S3. Loss of Betalain expression cassette T-DNA (pX11) in plants 5-1, 3-1 and 5-2

Supports Figure 5 and Figure 6.

This type of deletion event of plant 5-1, 3-1 and 5-2 was detected only in a plant with Cas9 and where a DSB was induced and not in control plants. The distance between the DSB and the end of the T-DNA integration sites is 180,895 bp. F2 progeny of each of these plants that were homozygous to MT SNPS in the regions flanking the gRNA recognition site and the pX11 T-DNA insertion site, were analyzed by WGS. In the deleted region there were no reads.

A: In plant 5-1 a part of the pX11 T-DNA was lost. The region with a blue box behind it is the part missing in plant 5-1. The brown boxes with blue arrows in them are the NOS terminator repeated sequences in inverted orientations. These sequences could anneal and generate a loop of the sequence between them. The empty purple triangle indicates the pX11 cassette's sequences that are present in plant 5-1 but do not give the Betalain color. KanR: kanamycin resistance gene; DODA1: *B. vulgaris* DOPA 4,5-dioxygenase; CYP76: *B. vulgaris* cytochrome P450; cDOPAgt: *M. jalapa* cyclo-DOPA-5-O-glucosyltransferase; Nos P/T: nopaline synthase promoter/terminator; 35S P/T: CaMV 35S promoter/terminator; AtAct2_T: Arabidopsis *ACTIN2* terminator; Ubi10_P: Arabidopsis *UBIQUITIN10* promoter.

B: In plants 3-1 and 5-2, two independent F1 regenerated green plants, the same region of MT chromosome 11 was deleted. A1 and A2 are A rich repeats flanking the deleted region.

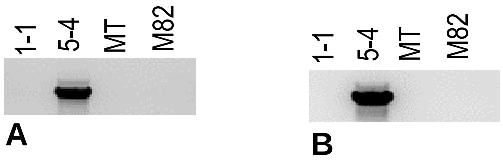

Supplemental Figure S4. PCR amplification of chromosome 11 translocation into chromosome 9 junctions from plant 5-4.

Supports Figure 7.

Two PCR primers sets were used for amplification of the chromosome 11 translocation into chromosome 9 junctions (Supplementary Table S6.). In both cases, a PCR product specific to plant 5-4 was amplified.

A: Amplification of the downstream junction with primers gRNA_pair2_far_F and CHR9_insert_CH11_GR2_R.

B: Amplification of the upstream junction with primers CH9_trans_US_F2 and gRNA_pair2_far_F.

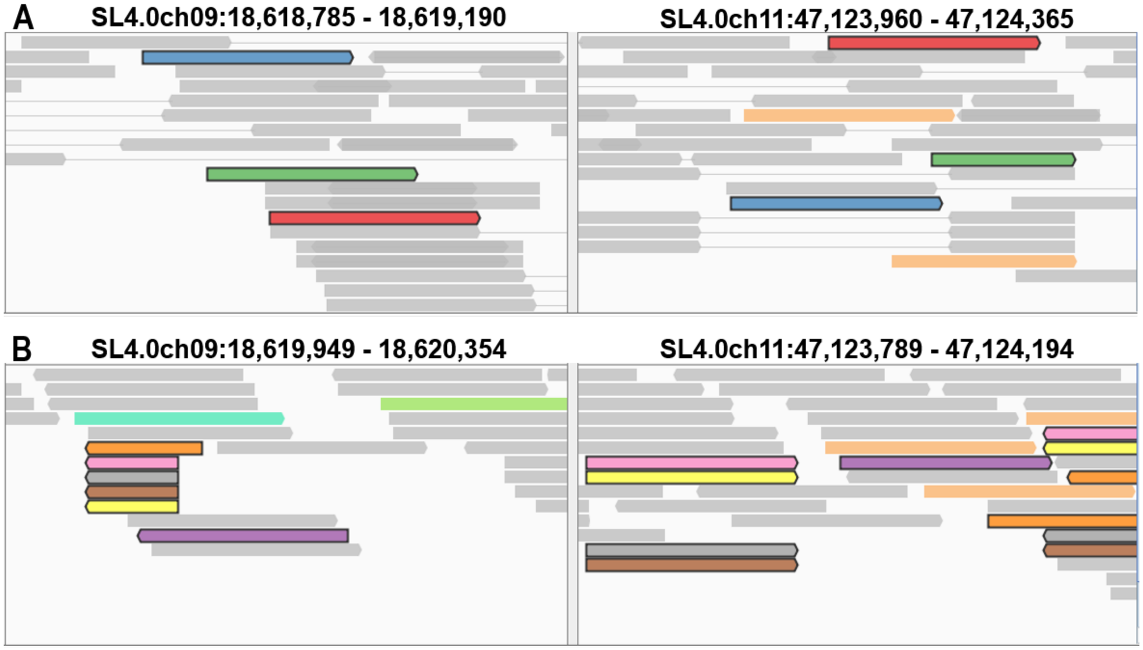

Supplemental Figure S5. Illumina reads of chromosome 11 translocation into chromosome 9 junctions.

Supports Figure 7.

IGV presentation of the Illumina reads of chromosome 11 translocation into chromosome 9 junctions in plant 5-4. Reads pairs with black lining and the same color indicate either pairs in which the two reads map to the two different chromosomes, or split reads in which a single read out of the two span the putative translocation site and thus map to two different chromosomes. A: Reads of the upstream junction of chromosome 9 and chromosome 11. In this case, no individual reads span the translocation site, while three read pairs are formed by individual reads mapping to chromosome 9 and chromosome 11. B: Reads of the downstream junction of chromosome 9 and chromosome 11. Here one read pair is formed by two individual reads falling entirely on chromosome 9 or chromosome 11 (purple), while five out of six reads span the translocation junction between the two chromosomes, thus appearing truncated and on both sides of the panel, i.e. both on chromosome 9 and 11. These reads therefore represent the transition from chromosome 9 to chromosome 11 sequences.

Primer name	Sequence	Primer code in Figure 2
6746R	GGCAAGATTAATCCAACTGGCAA	A1
7454F	ATTTTCCACCATGATATTCGGCAAG	A2
6653R	ATAAGGACGAGATGGTGGAGTAAAG	B1
8143F	GAACGTCAGTGGAGCATTTTTGA	B2

Supplemental Table S1. LB primers for inverse PCR. Supports Figure 2. Nested primers used for the inverse PCR of the T-DNA left border (LB). The first pair, 6746R + 7454F, is the inner pair equivalent to A1+A2 in Figure 2. The second pair, 6653R + 8143F, is the outer pair equivalent to B1+B2 in figure 2.

Target name	Sequence	Coordinates
PSY1 gRNA	AGCGTATATAATGCTGCTTTGG	SL4.0 Ch03: 4,236,695
gRNA2	AGACGACACTCAAAACAACAAGG	SL4.0 Ch11: 47,124,456

Supplemental Table S2. Cas9 DSB Targets on chromosome 3 and chromosome 11. Supports Figure 2. The sequence and coordinates for both targets putative DSB sites. PAM sequence (NGG) in each target is marked in red.

Primer name	Sequence
Cas9 long_F	CAGAATGAGAAGCTCTACCTCTACTACCTC
Cas9 long_R	GAAATTCATGATGTTAGAGTAGAAGAAATACTTAG

Supplemental Table S3. PCR Primers used for SpCas9 positive plants screening and selection. Supports Figure 3 and Figure 4. SpCas9 primers for verification of Cas9 T-DNA cassette presence in transgenic plants.

	**Chromosome 3 *PSY1* gRNA**				**Chromosome 11 gRNA2**			
	**M82/MT F1 in greenhouse**		**M82/MT F1 in tissue culture**		**M82/MT F1 in greenhouse**		**M82/MT F1 in tissue culture**	
	**SpCas9**	**SpCas9 +gRNA**	**SpCas9**	**SpCas9 +gRNA**	**SpCas9**	**SpCas9 +gRNA**	**SpCas9**	**SpCas9 +gRNA**
**Number of plants**	10	10	10	10	10	10	12	9
**Number of silencing events**	0	0	1	2	0	0	1	1
**Number of LOH T-DNA loss events**	0	0	0	0	0	0	0	3
**Number of LOH chromosome arm loss**	0	0	0	0	0	0	0	2
**Number of LOH whole chromosome loss**	0	0	0	0	0	0	0	1
**Number of LOH crossover**	0	1	0	0	0	0	0	0

Supplemental Table S4. Silencing and LOH events frequencies. Supports Figure 3, Figure 4, Figure 5 and Figure 6. Number of silencing and LOH events in chromosome 3 or chromosome 11. Plants grown in the greenhouse or cut and regenerated in tissue culture. Control plants with SpCas9 only, and treatment plants with SpCas9 + gRNA.

**Primer pair name**	**Primer pair Sequences**	**SL4.0 SNP Coordinates**
Ch3_set8_seq_F/R	TGTGGGCTTCTCGGATTGAATG<br>ACAAATTTTTCTGTTTAAGTTGTTTTGGA	Ch03: 3899707
SlPSY1_HTS_F/R	GTATCGCCCCTGAATCAAAG<br>AGTTCTGCAATTTTATTCCCAG	Ch03: 4236695
Ch3_set9_F/R	GGTTTCCTTGTACTCCCTCCG<br>GAGCTGGCTGTTTGGTATTTGG	Ch03: 4368283<br>Ch03: 4368284<br>Ch03: 4368296
Ch11_set8_F/R	CAACGCCTTGTGGTCTCTCT<br>TGCATTTCAGGCTTTTAGTGGT	Ch11: 37342774
Ch11_set2_F/R	TGCCTACCAGAGTCATATTTAGCC<br>TTGCATCTCGTTGGTCGATGT	Ch11: 43910448
gRNA_sanger_pair2_F/R	TCTCCACACCAGTCAATGGT<br>GGCATGGCTTGATTACGAAAGG	Ch11: 47124456
Ch11_set14_F/R	CAAAGAAGCTCCAACAGACATTCAA<br>CATGACGATTTGACCTAAAGGGTTT	Ch11: 47417708<br>Ch11: 47417713
Ch11_set10_F/R	AGTATCCAACTATTCAAGTTCCTCT<br>CCTCAGGGACTAGCATTATCTCT	Ch11: 47419506<br>Ch11: 47419597<br>Ch11: 47420107<br>Ch11: 47420578
Ch11_set11_F/R	AGTCATCCATTAAAGCACTCAAAA<br>TGTGAATGGTACTTAGACAAGAACT	Ch11: 47423702
clv3-conf-F/R	CGTGAGTCTTTACTGCCCTGT<br>GGGCCAAAAACAACAAAAAC	Ch11: 52945155

Supplemental Table S5. Primers used for sequencing of SNPs in F1, F2 and F3 plants. Supports Figure 3 and Figure 5. Each primer pair was used for PCR amplification of the SNP region. One or both primers of each set were used for Sanger sequencing of the PCR amplicon.

Primer pair name	Primer pair Sequences	Primer code in Figure 2
Ch11_gR2_long_F	GCCAATGAGTCAGGGGCAGAGCCAGCATAGTATTCTTAGGAAGTCAAGAAAATATTACCAGTGAC	A1
Solyc11g062260_promoter_F	CCGACAATGCGCGACTCCAGACACCGGGTAGGAAACCA	A2
Seq8_long_F	GTGAATGATTGTGAGTGTGGAAGAGAACAAAAATTGTCGTGCAATGCGCGCAAGG	B1
Solyc11g062260_5UTR_F	GGTGAAGTTTTGGTTTTTATAAGCAAATGTGGCGTTAATTGCTTCCTGATTGTTGTTGCGATCCG	B2

Supplemental Table S6. Primers used for inverse PCR and detection of plant 5-4 chromosome 11 into chromosome 9 translocation junctions. Supports Figure 7. Nested primers used for the inverse PCR and detection of plant 5-4 chromosome 11 into chromosome 9 translocation. The first pair, Ch11_gR2_long_F + Solyc11g062260_promoter_F, is the inner pair equivalent to A1+A2 in figure 2. The second pair, Seq8_long_F + Solyc11g062260_5UTR_F, is the outer pair equivalent to B1+B2 in Figure 2.

Primer pair name	Primer pair Sequences	SL4.0 Coordinates
gRNA_pair2_far_F	CGCTCCGCCACTAAACTAGA	Ch11: 47123478- 47123497
CHR9_insert_CH11_GR2_R	TGTTAAACGATAGACCCAACCGA	Ch09: 18620565-18620543
CH9_trans_US_F2	CACCATGGTTTAAGGGTCACCT	Ch09: 18619155- 18619176

Supplemental Table S7. Primers used for sequencing of plant 5-4 chromosome 11 into chromosome 9 translocation junctions. Supports Figure 7. Each primer pair was used for PCR amplification of the junction region. One or both primers of each set were used for Sanger sequencing of the PCR amplicon. gRNA_pair2_far_F is on chromosome 11 side of both junctions, and was paired with each of the chromosome 9 primers.

Plant	Plant number	Number of reads (x million)	Coverage	Library Complexity (% of duplicates)
Chr 3 CO	F2 124	33.2	X12	14%
Chr11 T-DNA loss	3-1 (F2 22)	13.7	X5	9%
Chr11 T-DNA partial loss (LB, KanR, DODA)	5-1(F2 10)	81.4	X31	7%
Chr11 T-DNA loss	5-2 (F2 24)	24.6	X9	11%
Chr11 arm loss	1-1	17.9	X6	5%
Chr11 arm loss	5-4	28.9	X11	12%
Chr11 loss	9-1	43.6	X16	15%
MT		65.5	X25	
M82		55	X21	18%

Supplemental Table S8. Whole genome sequencing (WGS) coverage. Supports Figure 3 and Figure 6.For WGS we used Illumina NovaSeq 6000, with 150 bp paired-end reads.

Coverage was calculated as read length (300 bp) x number of reads / haploid genome length (Tomato SL4.0 = 782,520,133 bp).

Chr- chromosome, MT- Micro-Tom.
